# Long non-coding RNA CCDC144NL-AS1 sponges miR-143-3p and regulates MAP3K7 by acting as a competing endogenous RNA in gastric cancer

**DOI:** 10.1038/s41419-020-02740-2

**Published:** 2020-07-09

**Authors:** Hao Fan, Yugang Ge, Xiang Ma, Zengliang Li, Liang Shi, Linling Lin, Jian Xiao, Wangwang Chen, Peidong Ni, Li Yang, Zekuan Xu

**Affiliations:** 1 Department of General Surgery, the First Affiliated Hospital of Nanjing Medical University, Nanjing, Jiangsu Province China; 2 Department of General Surgery, Liyang People’s Hospital, Liyang Branch Hospital of Jiangsu Province Hospital, Liyang, Jiangsu Province China

**Keywords:** Cancer, Diseases

## Abstract

Gastric cancer (GC) has been one of the most leading cause of cancer-death worldwide. Long non-coding RNAs (lncRNAs) have been found to be related with the carcinogenesis and the development of various cancers, including GC. However, there are still many GC-related lncRNAs functional roles and molecular mechanisms that have not yet been clearly studied. Herein, we report lncRNA CCDC144NL-AS1, which has not been explored in GC, and it is markedly upregulated in GC tissues, which may serve as an independent predictor of poor prognosis. We found that CCDC144NL-AS1 expression was significantly positively associated with a larger tumor size and more pronounced lymph node metastasis. Through a series of in vivo and in vitro functional experiments, we observed that CCDC144NL-AS1 could facilitate cell proliferation, invasion and migration and inhibit cell apoptosis in GC. Further mechanism investigation revealed that CCDC144NL-AS1 acted as a competing endogenous RNA (ceRNA) for sponging miR-143-3p and upregulated the expression of its direct endogenous target MAP3K7 in GC. Taken together, our results elucidate the oncogenic roles of CCDC144NL-AS1/miR-143-3p/MAP3K7 axis in GC progression, providing inspiration for further understanding of the mechanism of GC and making CCDC144NL-AS1 as a potential novel diagnostic and therapeutic target for GC.

## Introduction

Gastric cancer (GC) ranks among the highest malignancies, and it is also an important risk factor that endangers human health worldwide. Its morbidity and mortality are high, and the lack of effective treatments leads to a very poor prognosis^1,2^. Although in recent decades we have made some progress in fields such as surgical techniques, immunotherapy and molecular targeted therapy, the therapeutic effect is still not very satisfactory, which is attributed to the fact that we know little about the specific mechanism underlying the development of gastric carcinogenesis at the cellular and molecular level^3–5^. Therefore, it is significant meaningful to elucidate the molecular mechanism of the pathogenesis of GC and to find promising and effective diagnostic and therapeutic target for GC.

The Encyclopedia of DNA Elements (ENCODE) Project Consortium has revealed that the majority of the human genome are transcribed into non-coding RNAs (ncRNAs), while protein-coding genes account for only 2%^6^. Long non-coding RNAs (lncRNAs) share limited or no protein-coding capacity and have transcripts of more than 200 nucleotides in length^7^. LncRNAs usually play their biological functions by directly or indirectly regulating the expression of potential target genes at epigenetic modification, transcriptional and post-transcriptional levels^8,9^. These biological processes include cell proliferation, differentiation, and metastasis^10^. A growing number of studies show that dysregulation of lncRNAs are related with the development and the carcinogenesis of many tumors, including GC^11–14^. Recently, lncRNAs have been widely reported to function as competing endogenous RNA (ceRNA), ceRNA can affect gene silencing caused by microRNA through binding microRNA response elements (MREs), which reveals the existence of a RNA-microRNA regulatory pathway and has great biological significance^15–17^. Construction and analysis of lncRNA-mediated ceRNA network has opened a new way for understanding the pathogenesis of GC and finding novel diagnostic biomarkers or potential therapeutic targets for GC.

In the present study, we found a GC-associated lncRNA CCDC144NL-AS1, which was markedly upregulated in GC tissues and related with poor prognosis, while its function in GC has not been reported. Through a series of in vitro and in vivo functional experiments, we found that CCDC144NL-AS1 could facilitate cell proliferation, invasion and migration in GC. With the deepening of research, we discovered that CCDC144NL-AS1 might regulate MAP3K7 expression by sponging miR-143-3p to exert ceRNA function. Taken together, our study elucidates oncogenic roles of CCDC144NL-AS1/miR-143-3p/MAP3K7 axis in GC tumorigenesis, which may provide a prognostic marker as well as a promising therapeutic target for GC patients.

## Results

### CCDC144NL-AS1 is upregulated in GC and associated with poor prognosis

**Fig. 1:**
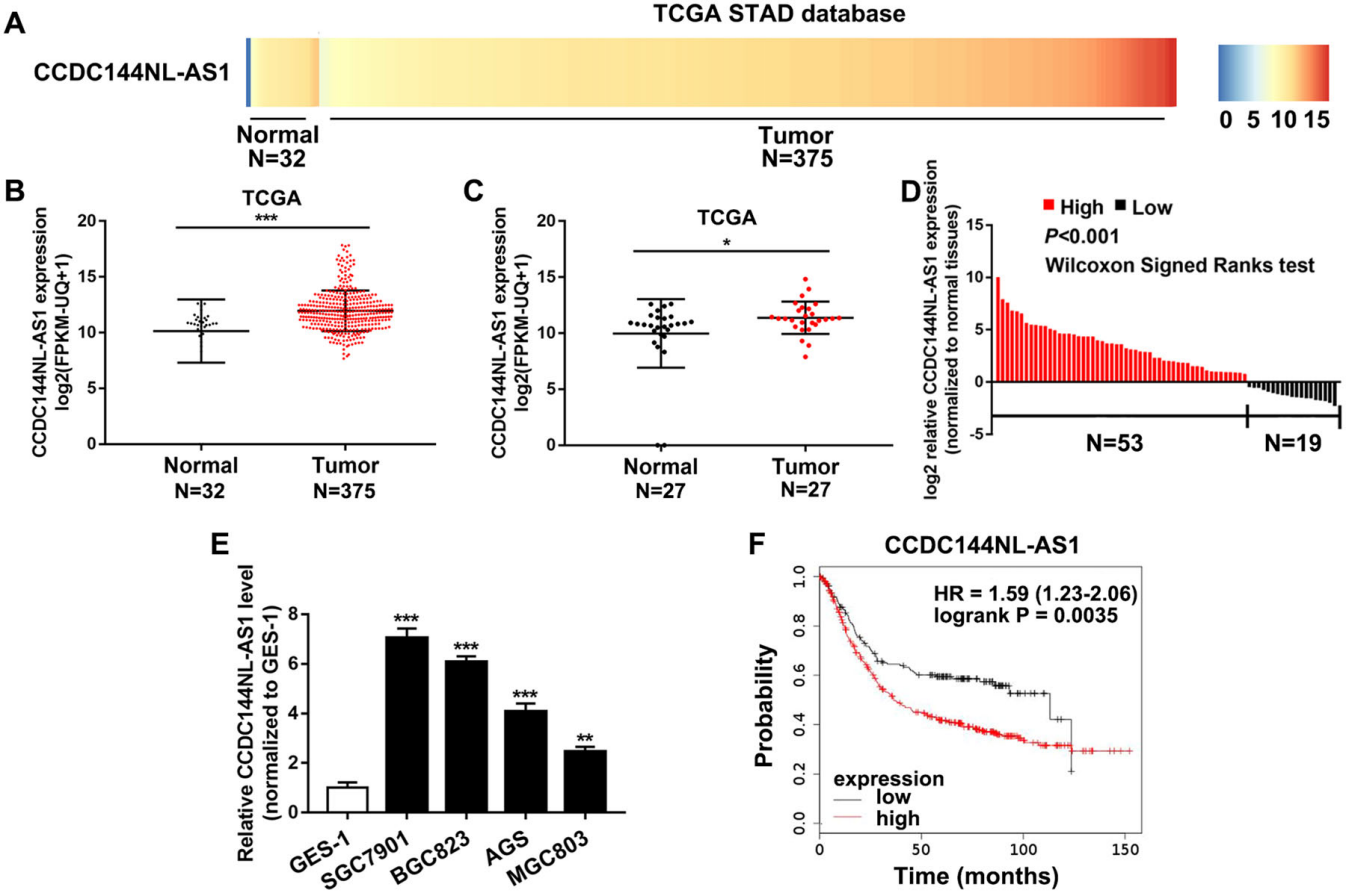
CCDC144NL-AS1 is upregulated in GC and associated with poor prognosis. **a** Heat map analysis of CCDC144NL-AS1 expression in GC from the TCGA database. **b**, **c** Relative expression of CCDC144NL-AS1 in unpaired and paired GC tissues was analyzed from the TCGA database. **d** qRT-PCR was used to detect the expression of CCDC144NL-AS1 in 72 GC tissues and paired adjacent normal tissues. **e** qRT-PCR analysis of CCDC144NL-AS1 expression in GC cells and normal gastric epithelium cell line (GES-1). **f** Online Kaplan–Meier overall survival (OS) curves according to CCDC144NL-AS1 expression levels. *p < 0.05, **p < 0.01, ***p < 0.001.

By analyzing TCGA STAD database including RNA sequencing (RNA-seq) data of 375 GC tissues and 32 adjacent non-tumor tissues, CCDC144NL-AS1 was identified as GC-associated lncRNA that may be related with gastric tumorigenesis. As a result, we took heat map analysis and found that the gene expression of CCDC144NL-AS1 was markedly elevated in GC patients, which suggested that CCDC144NL-AS1 might play huge roles in GC (Fig. 1a). We retrieved TCGA STAD database and discovered that CCDC144NL-AS1 mRNA expression was markedly overexpressed both in unpaired and paired GC tissues (Fig. 1b, c). Then, we detected the mRNA expression of CCDC144NL-AS1 in our own 72 paired GC tissues and cell lines, which suggested a high expression of CCDC144NL-AS1 in GC (Fig. 1d, e). SGC7901 and BGC823 cell lines with the highest CCDC144NL-AS1 expression levels were selected for subsequent experiments. The clinicopathologic features of the GC patients showed that high CCDC144NL-AS1 expression was obviously related with a larger tumor size and more profound lymphatic metastasis rate (Table S1).Further, the online Kaplan–Meier plot (http://www.kmplot.com) indicated that a high CCDC144NL-AS1 expression level was correlated with poor overall survival (Fig. 1f). These results revealed that CCDC144NL-AS1 was upregulated in GC and related with poor prognosis.

### Altering the expression of CCDC144NL-AS1 affects the proliferation, migration and invasion of GC cells

**Fig. 2:**
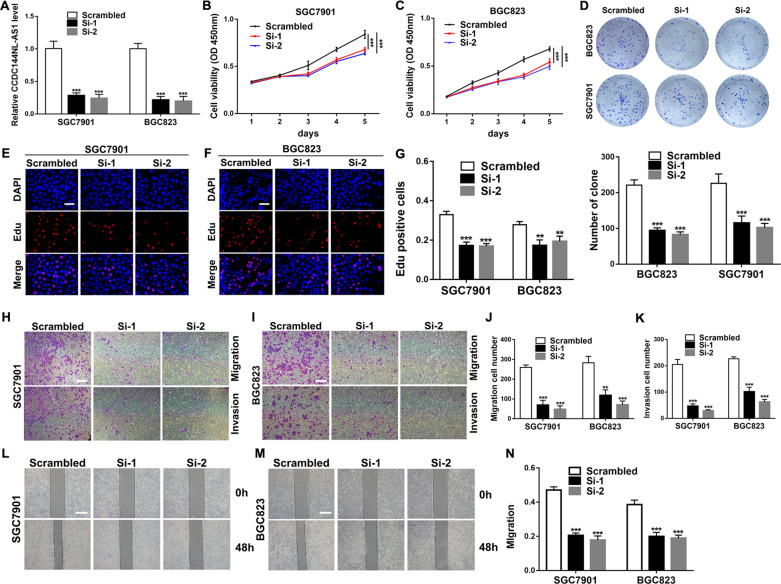
Knockdown of CCDC144NL-AS1 inhibits GC cell proliferation, migration, and invasion. **a** qRT-PCR analysis of CCDC144NL-AS1 mRNA in SGC7901 and BGC823 cells treated with si-CCDC144NL-AS1. **b**, **c** CCK-8 assay was applied to detect cell viability. **d**–**g** Colony formation assays and the Edu assays were conducted to measure cell proliferation ability in BGC823 and SGC7901 cells (scale bar: 100 μm for Edu assay). **h–n** Representative results of transwell assays and wound healing assays of GC cells after si-CCDC144NL-AS1 transfection (scale bar: 200 μm for transwell assay, 100 μm for wound healing assay). **p < 0.01, ***p < 0.001.

To explore the biological functions of CCDC144NL-AS1 in GC cells, we knocked down CCDC144NL-AS1 expression by transfecting with siRNAs in BGC823 and SGC7901 cells (Fig. 2a). CCK-8 proliferation experiments suggested that silencing CCDC144NL-AS1 could dramatically attenuate cell proliferation ability compared with the negative control (NC) (Fig. 2b, c). Colony formation assay indicated that downregulation of CCDC144NL-AS1 significantly reduced the cell colony ability of SGC7901 and BGC823 cells (Fig. 2d). Likewise, Edu assays showed the same results that CCDC144NL-AS1 silencing inhibits GC cell proliferation (Fig. 2e–g). Furthermore, transwell assays demonstrated that CCDC144NL-AS1 knockdown repressed the migration and invasion of GC cells (Fig. 2h–k). Moreover, in the wound healing assays, silencing CCDC144NL-AS1 attenuated the migration rate of GC cells (Fig. 2l–n).

In addition, we upregulated CCDC144NL-AS1 by transfecting pcDNA-CCDC144NL-AS1 into SGC7901 and BGC823 cells and the expression level of CCDC144NL-AS1 increased (Fig. S1A). CCK-8 assays, colony formation assays and Edu assays revealed that overexpression of CCDC144NL-AS1 could promote SGC7901 and BGC823 cells proliferation (Fig. S1B–G). Furthermore, transwell assays suggested that the cell numbers of migrated and invaded cells increased by overexpressing CCDC144NL-AS1 (Fig. S1H–K). Wound healing assays showed the same results as transwell migration assays (Fig. S1L–N). These results suggested that silencing CCDC144NL-AS1 inhibited the proliferation, migration and invasion of GC cells and overexpression of CCDC144NL-AS1 had the opposite effects. CCDC144NL-AS1 may play a key role as an oncogene in GC.

### Knockdown of CCDC144NL-AS1 induces cell apoptosis, inhibits GC tumor growth and metastasis in vivo

**Fig. 3:**
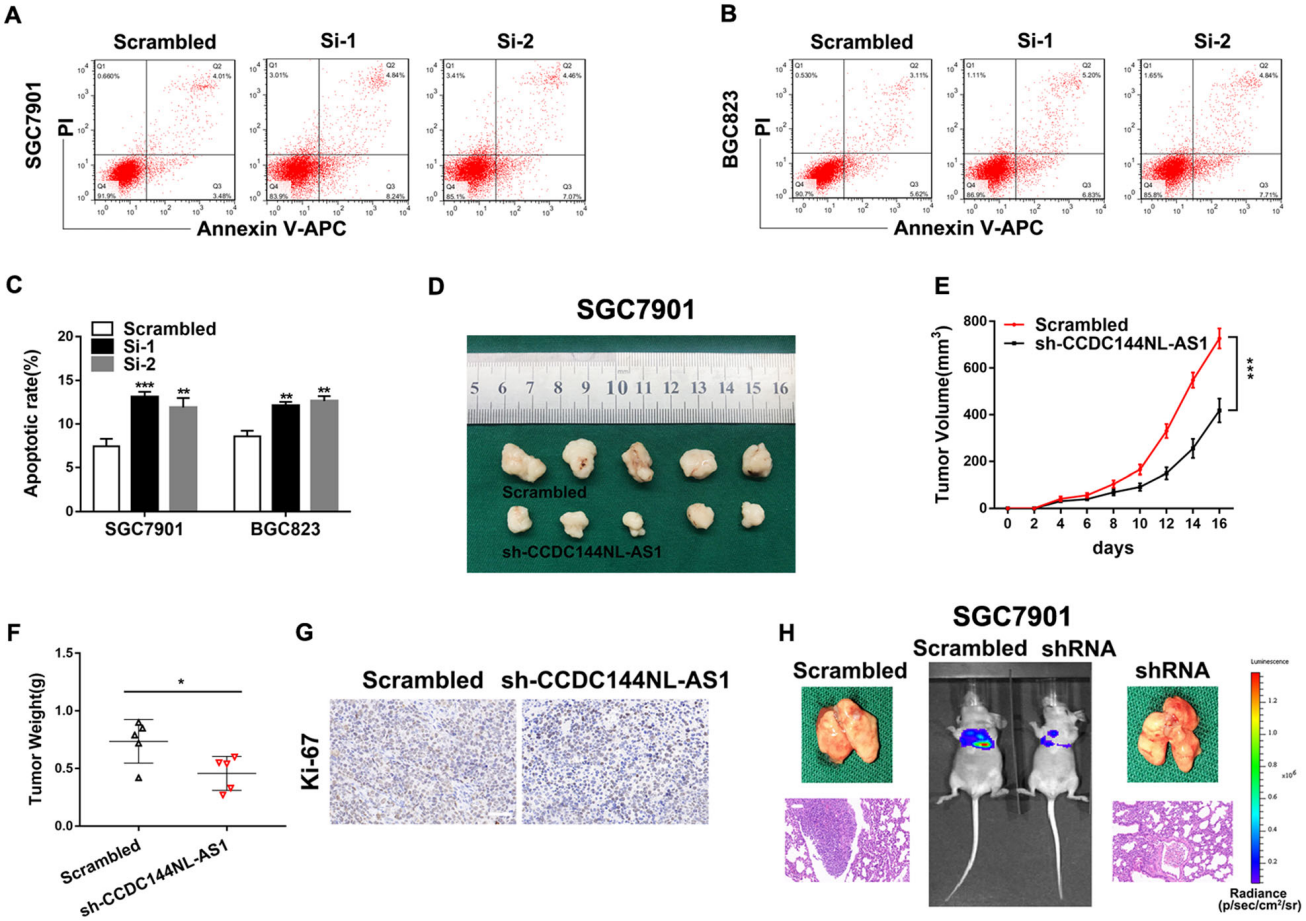
Knockdown of CCDC144NL-AS1 induces cell apoptosis, inhibits GC tumor growth, and metastasis in vivo. **a**–**c** Flow cytometry analysis of BGC823 and SGC7901 cells apoptosis rates. **d** Xenograft tumors in the nude mouse model under different treatments. **e**, **f** Analysis of tumor size and weight in different groups. **g** The expression of Ki-67 from the xenografts were measured by IHC (scale bar: 50 μm). **h** Representative images of lung metastasis and HE staining of specimen (scale bar: 50 μm). *p < 0.05, **p < 0.01, ***p < 0.001.

The cell apoptosis is an important factor which could influence GC cell proliferation. Flow cytometric assays revealed that knockdown of CCDC144NL-AS1 induced SGC7901 and BGC823 cells apoptosis (Fig. 3a–c). Subsequently, we inoculated subcutaneously the SGC7901 cells stably transfected with shRNA targeting CCDC144NL-AS1 or their negative control groups into female nude mice to determine whether CCDC144NL-AS1 affected tumor growth in vivo, we discovered that the average tumor size and weight generated by CCDC144NL-AS1 knockdown group were markedly smaller than those generated from their control group (Fig. 3d–f). Tumor tissues harvested from nude mice was used to detect the expression level of proliferation marker Ki-67 by IHC staining, it suggested that the Ki-67 expression was lower in sh-CCDC144NL-AS1 group than control group (Fig. 3g). To explore the function of CCDC144NL-AS1 on tumor metastasis in vivo, stable transfected cells treated differently were injected into tail vein of BALB/c nude mice separately. The results suggested that lung metastasis in CCDC144NL-AS1 knockdown group was alleviated (Fig. 3h). These results showed that silencing CCDC144NL-AS1 induced cell apoptosis, inhibited GC tumor growth and metastasis in vivo.

### CCDC144NL-AS1 acts as a molecular sponge for miR-143-3p in GC cells

**Fig. 4:**
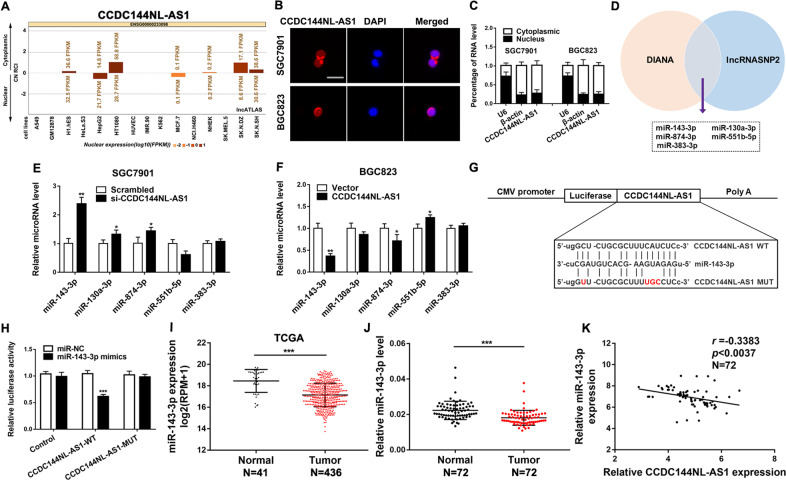
CCDC144NL-AS1 acts as a molecular sponge for miR-143-3p in GC cells. **a** Prediction of CCDC144NL-AS1 localization in cells by bioinformatics tools. **b**, **c** Subcellular localization of CCDC144NL-AS1 in GC cells was analyzed by FISH and qRT-PCR (scale bar: 50 μm for FISH assay). **d** DIANA and lncRNASNP2 databases predict miRNAs binding to CCDC144NL-AS1. **e**, **f** The relative expressions of miRNAs in GC cells after upregulation or downregulation of CCDC144NL-AS1. **g** The predicted binding sites between CCDC144NL-AS1 and miR-143-3p. **h** miR-143-3p mimics markedly reduced luciferase activity in CCDC144NL-AS1-wild not in CCDC144NL-AS1-mut in HEK-293T cells. **i** Relative expression of miR-143-3p in GC tissues and adjacent normal tissues was analyzed from the TCGA database. **j** qRT-PCR was used to detect miR-143-3p expression in 72 GC tissues and paired adjacent normal tissues. **k** Correlation analysis of the expression of CCDC144NL-AS1 and miR-143-3p in 72 GC tissues. *p < 0.05, **p < 0.01, ***p < 0.001.

To explore how CCDC144NL-AS1 exerts its function, we predicted its subcellular localization by lncATLAS (http://lncatlas.crg.eu/). The result showed that CCDC144NL-AS1 was mainly located in the cytoplasm of all the available cell types (Fig. 4a). FISH assay and quantitative RT-PCR analysis suggested that CCDC144NL-AS1 was mainly located in the cytoplasm of GC cells (Fig. 4b, c). These results indicated that CCDC144NL-AS1 may function as a ceRNA of miRNAs. Then, we predicted the potential miRNAs that may interact with CCDC144NL-AS1 using DIANA (http://carolina.imis.athena-innovation.gr/diana_tools/web/index.php?r=site%2Ftools) and lncRNASNP2 (http://bioinfo.life.hust.edu.cn/lncRNASNP/#!/) database and observed that CCDC144NL-AS1 sequence contains potential binding sites of several miRNAs such as miR-143-3p, miR-130a-5p, miR-874-3p, miR-551b-5p, miR-383-3p (Fig. 4d). We then explored the effect of downregulation or upregulation of CCDC144NL-AS1 on the expression level of these miRNAs and found that miR-143-3p was the most changed one (Fig. 4e, f). A dual-luciferase reporter assay showed that miR-143-3p significantly reduced the relative luciferase activity of the wild-type CCDC144NL-AS1 3’UTR (Fig. 4g, h). TCGA database has revealed that miR-143-3p was downregulated in GC tissues, which suggested that miR-143-3p might function as a tumor suppressor gene (Fig. 4i). Consistent with TCGA analysis results, we verified lower miR-143-3p expression in 72 GC tissues (Fig. 4j). A negative correlation was also found between CCDC144NL-AS1 and miR-143-3p expression in 72 pairs of GC samples (Fig. 4k). These results indicated that CCDC144NL-AS1 directly “sponges” miR-143-3p.

### The regulation of CCDC144NL-AS1 on GC cells is mediated by miR-143-3p

**Fig. 5:**
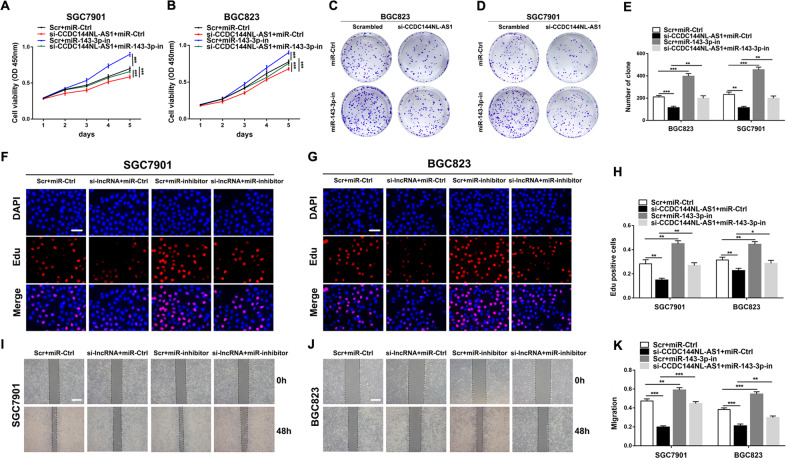
The regulation of CCDC144NL-AS1 on GC cells is mediated by miR-143-3p. **a**–**h** CCK-8 assays, colony formation assays and the Edu assays were used to detect the cell proliferation ability after transfecting GC cells with scrambled, si-CCDC144NL-AS1, miR-143-3p inhibitor or si-CCDC144NL-AS1+miR-143-3p inhibitor (scale bar: 100 μm for Edu assay). **i**–**k** The wound healing assays were used to detect the cell migration ability after transfecting GC cells (scale bar: 100 μm). *p < 0.05, **p < 0.01, ***p < 0.001.

To further explore the biological interactions between CCDC144NL-AS1 and miR-143-3p in GC, SGC7901, and BGC823 cells were co-transfected with si-CCDC144NL-AS1 and miR-143-3p inhibitor. From CCK-8 assays, colony formation assays, Edu assays, and wound healing assays, we observed that knockdown of CCDC144NL-AS1 mediated inhibition of GC cell proliferation and migration were partially rescued by co-transfection with a miR-143-3p inhibitor (Fig. 5a–k). These results suggested that the carcinogenic effect of CCDC144NL-AS1 was partially mediated by negative regulation of miR-143-3p.

### MAP3K7, a target gene of miR-143-3p, is regulated by CCDC144NL-AS1

**Fig. 6:**
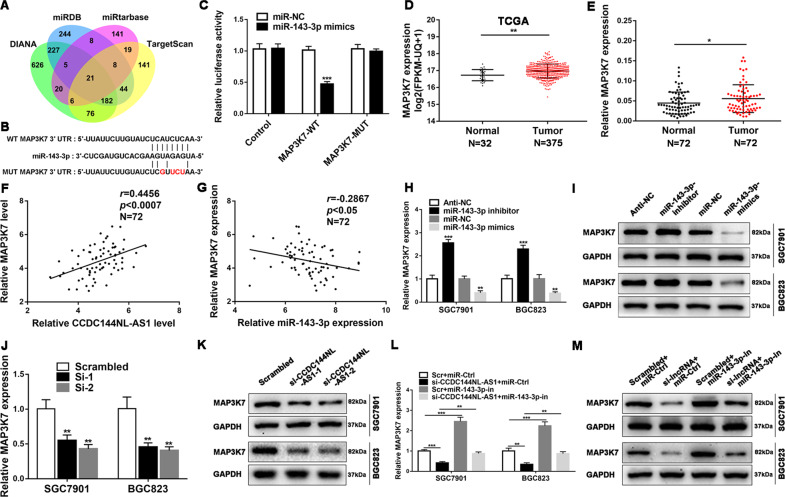
MAP3K7, a target gene of miR-143-3p, is regulated by CCDC144NL-AS1. **a** Predicted binding targets for miR-143-3p binding target via Diana, miRDB, Targetscan, and miRtarBase. **b** The predicted binding sites between MAP3K7 and miR-143-3p. **c**. miR-143-3p mimics prominently reduced luciferase activity in MAP3K7-wild not in MAP3K7-mut in HEK293T cells. **d** Relative expression of MAP3K7 in GC tissues compared with adjacent normal tissues was analyzed from the TCGA database. **e** qRT-PCR was used to detect the MAP3K7 expression in 72 GC tissues and paired adjacent normal tissues. **f**, **g** Correlation analysis of the expression of CCDC144NL-AS1, miR-143-3p and MAP3K7 in 72 GC cases. **h**, **i** Relative mRNA and protein levels of MAP3K7 in BGC823 and SGC7901 cells transfected with miR-143-3p inhibitor, miR-143-3p mimics or their control groups. **j**, **k** Relative mRNA and protein levels of MAP3K7 in GC cells transfected with si-CCDC144NL-AS1 or its scrambled control. **l**, **m** Relative mRNA and protein levels of MAP3K7 in GC cells transfected with scrambled, si-CCDC144NL-AS1, miR-143-3p inhibitor or si-CCDC144NL-AS1 + miR-143-3p inhibitor. *p < 0.05, **p < 0.01, ***p < 0.001.

To explore the ceRNA network between CCDC144NL-AS1, miR-143-3p, and its targets in GC, we used DIANA (http://diana.imis.athena-innovation.gr/DianaTools/index.php), miRDB (http://www.mirdb.org/), miRTarBase (http://mirtarbase.mbc.nctu.edu.tw/php/index.php), TargetScan (http://www.targetscan.org/vert_71/) to predict putative targets of miR-143-3p, 21 genes may be the direct targets of miR-143-3p (Fig. 6a). Based on TCGA database, we selected four genes with relative higher expression in GC than in normal tissues as candidate target genes (Fig. S2A). Western blot indicated that MAP3K7 protein level changed most obviously in SGC7901 cells transfected with miR-143-3p mimics or NC (Fig. S2B, C). A ceRNA network was constructed by Cytoscape (version 3.7.1) according to the data predicted by the above bioinformatics tools, in the network, we found that CCDC144NL-AS1 was indeed correlated the predicted miR-143-3p and MAP3K7 (Fig. S3). So, MAP3K7 was identified as a potential target of miR-143-3p. A dual-luciferase reporter assay revealed that miR-143-3p markedly reduced the relative luciferase activity of the wild-type MAP3K7 3′UTR (Fig. 6b, c). TCGA database revealed that MAP3K7 was upregulated in GC samples (Fig. 6d). Consistent with TCGA analysis results, we verified higher MAP3K7 expression in 72 GC tissues (Fig. 6e). A negative correlation was observed between MAP3K7 and miR-143-3p expression in 72 pairs of GC samples and a positive correlation was found between MAP3K7 and CCDC144NL-AS1 expression (Fig. 6f, g). Subsequently, we transfected SGC7901 and BGC823 cells with miR-143-3p mimics, miR-143-3p inhibitor and their negative control group to investigate whether MAP3K7 is regulated by miR-143-3p, we observed that the MAP3K7 mRNA and protein level changed with upregulation or downregulation of miR-143-3p, respectively (Fig. 6h, i, Fig. S4A). Further investigations indicated that downregulation of CCDC144NL-AS1 positively affected MAP3K7 expression both at the mRNA and protein levels (Fig. 6j, k, Fig. S4B). For the rescue experiment, knockdown of miR-143-3p counteracted the corresponding decrease in MAP3K7 mRNA or protein expression induced by downregulation of CCDC144NL-AS1 in SGC7901 and BGC823 cells (Fig. 6l, m, Fig. S4C). These results showed that MAP3K7 expression was primarily mediated by the regulation of miR-143-3p via CCDC144NL-AS1 at post-transcriptional level.

### Silencing MAP3K7 inhibits cell proliferation, induces GC cell apoptosis

To explore the potential role of MAP3K7 in GC, we transfected SGC7901 and BGC823 cells with siRNA to knockdown MAP3K7, which was identified at the mRNA and protein levels (Figs. S5A, B and S4D). CCK-8 assays and Edu assays indicated that knockdown of MAP3K7 could inhibit GC cell proliferation (Fig. S5C–G). To identify whether MAP3K7 knockdown could regulate tumor growth in vivo, SGC7901-shMAP3K7 and SGC7901-NC cells were subcutaneously inoculated into nude mice. We observed that the average size and weight of tumors generated by MAP3K7 knockdown group were markedly smaller (Fig. S5H–J). Flow cytometric assays also showed that knockdown of MAP3K7 induced GC cells apoptosis (Fig. S5K, L). These results suggested that silencing MAP3K7 inhibited cell proliferation, induced GC cell apoptosis.

### The regulation of MAP3K7 on GC cells is mediated by miR-143-3p

**Fig. 7:**
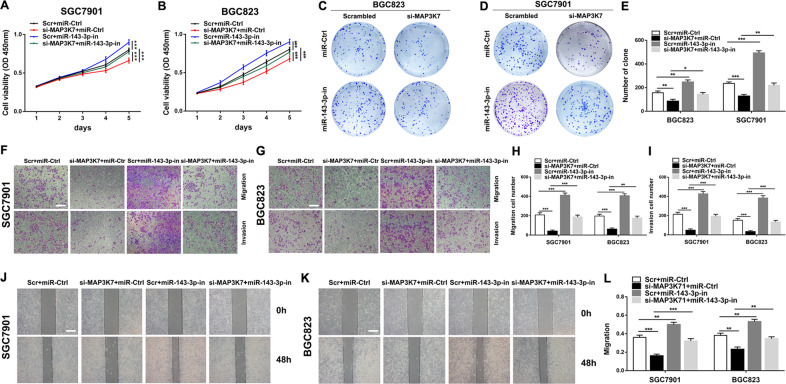
The regulation of MAP3K7 on GC cells is mediated by miR-143-3p. **a**–**e** CCK-8 assays and colony formation assays were used to detect the cell proliferation ability after transfecting GC cells with scrambled, si-MAP3K7, miR-143-3p inhibitor or si-MAP3K7 + miR-143-3p inhibitor. **f–l** Transwell assays and wound healing assays were used to detect the cell migration and invasion ability after transfecting GC cells (scale bar: 200 μm for transwell assay, 100 μm for wound healing assay). *p < 0.05, **p < 0.01, ***p < 0.001.

To further explore the biological interactions between MAP3K7 and miR-143-3p in GC, SGC7901, and BGC823 cells were co-transfected with si-MAP3K7, miR-143-3p inhibitor and their negative control groups. From CCK-8 assays, colony formation assays, transwell assays and wound healing assays, we observed that knockdown of MAP3K7 mediated inhibition of GC cell proliferation, migration and invasion were partially rescued by co-transfection with a miR-143-3p inhibitor (Fig. 7a–l). These results suggested that the carcinogenic effect of MAP3K7 was partially mediated by negative regulation of miR-143-3p.

## Discussion

Large numbers of lncRNAs have been found to take part in the progression of tumor, including GC^11,18,19^. However, there are still many GC-related lncRNAs functional roles and molecular mechanisms that have not yet been clearly investigated. In the present study, we first reported lncRNA CCDC144NL-AS1, which was highly expressed in GC tissues and cell lines. The high expression of CCDC144NL-AS1 was related to larger tumor size and lymphatic metastasis. Besides, increased CCDC144NL-AS1 expression was associated with shorter OS time of GC patients. Further analysis indicated that silencing CCDC144NL-AS1 could inhibit cell proliferation, migration and invasion in GC, and overexpression of CCDC144NL-AS1 yielded the opposite result. Meanwhile, in vivo experiments revealed that knockdown of CCDC144NL-AS1 alleviated lung metastasis and inhibited GC tumor growth. These results suggest that CCDC144NL-AS1 may exert a key function as an oncogene in GC.

The ceRNA hypothesis has attracted much attention in recent years^15^. Accumulating evidences suggests that lncRNA may act as a ceRNA for particular miRNAs to modulate the target genes of the miRNAs, including GC^16,17,20^. However, so far CCDC144NL-AS1 has never been reported to act as a ceRNA of miRNAs. In this study, we assessed the subcellular localization of CCDC144NL-AS1 and observed that majority of CCDC144NL-AS1 were located in cytoplasm, which suggested that CCDC144NL-AS1 may function as a ceRNA for sponging miRNA. The subsequent bioinformatics analyses and luciferase reporter assays revealed that CCDC144NL-AS1 may bind with miR-143-3p. miR-143-3p has been reported to be downregulated in many tumors and functioned as a tumor suppressor, including GC^21–23^. We also found that miR-143-3p was markedly downregulated in GC tissues, and its expression level was negatively correlated with CCDC144NL-AS1. Besides, downregulation of miR-143-3p could partially rescue the inhibition of GC cell proliferation mediated by knockdown of CCDC144NL-AS1. So we think that CCDC144NL-AS1 may play a huge role in GC cells by sponging miR-143-3p during GC progression.

Generally, as a ceRNA, the function of lncRNAs depends on the miRNA target. Therefore, the target genes of miRNA are an important part of the ceRNA network^24,25^. MAP3K7 was selected as a direct target of miR-143-3p through bioinformatics analyses and luciferase reporter assays, which has never been reported. MAP3K7, which is known as TAK1(TGF-β-activated kinase-1), can be rapidly activated in response to TGF-β signal transduction^26^. MAP3K7 has been reported to participate in the development of various tumors^27,28^. With the deepening of research, we found that the expression of MAP3K7 was upregulated in GC samples. A negative or positive correlation was found among MAP3K7, miR-143-3p and CCDC144NL-AS1 expression in GC samples. Knockdown of MAP3K7 could inhibit cell proliferation, induces GC cell apoptosis. Besides, rescue experiments revealed that miR-143-3p could change proliferation, migration and invasion capabilities of tumor cells by interacting with MAP3K7 and exhibit regulative effects on GC. Thus, these results implied that CCDC144NL-AS1 functioned as a molecular sponge for miR-143-3p and upregulated the expression of its endogenous target MAP3K7 in GC. However, our study also has some limitations, the interaction between cytokines in cancer cells is very complex, we cannot rule out the role of CCDC144NL-AS1 in promoting GC through other pathways. We studied this signaling axis only in the GC cell lines SGC7901 and BGC823, it is quite necessary to extend relevant researches to other GC cell lines.

In summary, we identified a novel GC-associated lncRNA CCDC144NL-AS1. Through integrating clinical data and multipronged functional experiments, we illustrated that CCDC144NL-AS1 was upregulated in GC tissues and associated with poor prognosis. CCDC144NL-AS1 upregulated MAP3K7 to promote GC cell proliferation, migration, and invasion by acting as a ceRNA that sponges miR-143-3p. Our results provide inspiration for further understanding of the mechanism of GC. In the future work, we will explore the mechanism of CCDC144NL-AS1 upregulation in GC, the correlation between CCDC144NL-AS1 and other miRNAs or proteins, which will further deepen our understanding of the pathogenesis of GC and make CCDC144NL-AS1 a potential novel diagnostic and therapeutic target for GC.

## Materials and methods

### Public data analysis

A TCGA gene dataset named TCGA-STAD.htseq_fpkm-uq.tsv (including 372 GC tissues and 35 normal tissues) with version number 07-20-2019 and A TCGA gene dataset named TCGA-STAD.mirna.tsv (including 436 GC tissues and 41 normal tissues) with version number 07-20-2019 were downloaded from the UCSC cancer browser (https://xenabrowser.net/datapages/). We obtained the normalized expression values of CCDC144NL-AS1, MAP3K7, and miR-143-3p from the above two files.

We have previously predicted miRNAs (miR-143-3p, miR-130a-3p, miR-874-3p, miR-551b-5p, and miR-383-3p) that bind to CCDC144NL-AS1 and showed 21 mRNAs targeted by miR-143-3p. In order to build the ceRNA network, we used R software to predict the target mRNAs of remaining four miRNAs through the data from the following four databases: DIANA, miRDB, miRTarBase, and TargetScan. Finally, the lncRNA-miRNA-mRNA regulatory network was constructed and visualized using Cytoscape software (version 3.7.1).

### Tissue specimens

A total of 72 GC tissues and matched normal tissues were obtained from patients with GC diagnosed by histopathological examination in the General Surgery Department of the First Affiliated Hospital of Nanjing Medical University (NMU). Immediately after excision, all samples were frozen with liquid nitrogen and stored at −80 °C until further use. This study was approved by the Ethics Committee of the First Affiliated Hospital of NMU. Written informed consent was signed before specimen collection.

### Cell culture

Human GC cell lines (SGC7901, BGC823, AGS, MGC803) and the human normal gastric epithelial cell line GES-1 were purchased from the Institute of Biochemistry and Cell Biology of the Chinese Academy of Sciences (Shanghai, China). SGC7901, BGC823, MGC803 and AGS are all gastric adenocarcinoma cells. SGC7901 originated from lymph node metastasis in a 56-year-old woman with gastric adenocarcinoma. BGC823 was built from a 62-year-old patient with gastric cancer and is poorly differentiated. MGC803 was established from a 53-year-old male patient with primary gastric poorly differentiated mucinous adenocarcinoma. AGS cell line originated from an untreated excised tumor fragment (http://www.cellbank.org.cn/search.asp?a=1). SGC7901, BGC823, MGC803 and GES-1 cells were maintained in RPMI-1640 medium (Gibco, USA), AGS cells were cultured in F12K medium (Gibco, USA), supplemented with 10% fetal bovine serum (FBS, Gibco, USA) at 37 °C in a 5% CO_2_ incubator.

### Quantitative real-time reverse transcription polymerase chain reaction (qRT-PCR)

LncRNA/mRNA was reverse-transcribed to cDNA with a Prime Script RT reagent Kit (Takara, China), a New Poly (A) Tailing Kit (Thermo Fisher Scientific, China) was used to reverse transcribe miRNAs into cDNA. cDNA was amplified with a 7500 Real-Time PCR System (Applied Biosystems, USA) with Universal SYBR Green Master Mix (Roche, Shanghai, China). β-actin or U6 was used as an endogenous control for mRNA and miRNA, respectively. All primer sequences are listed in Supplementary Table 2.

### Cell transfection

Transfections were carried out using the Lipofectamine 3000 Reagent (Invitrogen, USA) following the manufacturer’s protocol. The small interfering RNAs (siRNAs) targeting lncRNA/mRNA, miRNA mimics, miRNA inhibitors and their negative controls were designed and synthesized by GenePharma (Shanghai, China). Human CCDC144NL-AS1 transcript cDNA and short hairpin RNA (shRNA) directed against CCDC144NL-AS1 were cloned into the lentivirus vector CV084 and vector GV248, respectively (GenePharma, China). Stable cells were selected using puromycin. The sequences are listed in supplementary Table 2.

### Western blot

Proteins were extracted and electrophoresed on a 10% SDS polyacrylamide gels, and then electrophoretically transferred onto a polyvinylidene fluoride (PVDF) membrane (Millipore, USA). The ECL chemiluminescent reagent (Beyotime Biotechnology, China) was used to detect chemical signals. The antibodies are listed in supplementary Table 2.

### Cell counting kit-8 assay

Thousand cells per well were seeded into 96-well plate and the cell proliferation rate was measured using Cell counting kit-8 assay (CCK-8) (Djingo, Japan) for 5 days. The absorbance at 450 nm was recorded using a standard microplate reader (Scientific MultiskanMK3, Thermo Scientific) according to the manufacturers’ protocols.

### 5-Ethynyl-2′-deoxyuridine (Edu) assay

The Edu assay kit (RiboBio, China) was used to measure cell proliferation. Briefly, cells from different groups were treated with Edu and then stained by 4′,6-diamid-ino-2-phenylindole (DAPI). The images of Edu-positive cells were visualized under fluorescence microscope (Nikon, Japan).

### Colony formation

Cells who were in six-well plates were incubated at 37 °C in a 5% CO_2_ incubator for two weeks. Then cells were stained, imaged and counted for the number of colonies.

### Transwell assay

Transwell chambers with a membrane pore size of 8 mm were coated without or with Matrigel (BD Biosciences, USA). A total of 2 × 10^4^ cells were seeded into the upper chambers with serum-free medium, whereas medium containing 10% FBS was used in the lower chamber. After incubation for 24 h, the cells were fixed, stained, and counted using an inverted microscope.

### Wound healing assay

Cells were seeded in 6-well plates and cultured to the subfusion state. Linear scratch wounds were created by 200 μl sterile pipette tip and washed with phosphate-buffered saline (PBS). Images were captured at 0 and 48 h, we calculated cell healing rates by the fraction of cell coverage across the line.

### Animal experiments

Animal work was approved by the First Affiliated Hospital of Nanjing Medical University. For the tumor xenograft model, cells treated differently were subcutaneously inoculated into 4-week-old BALB/c nude mice (four or five mice for each group). Tumor volume was measured every 2 days. For about two weeks after injection, mice were euthanized and then the weight of xenografts was tested. For the metastasis model, cells treated differently were injected into the caudal vein of anesthetized nude mice (4 mice for each group). Mice were monitored using an IVIS imaging systems (Caliper Life Sciences, USA).

### Dual-Luciferase reporter assay

The putative miR-143-3p binding sequences in CCDC144NL-AS1 or MAP3K7-3ʹ-UTR and their mutant of the binding sites were synthesized and cloned to downstream of the luciferase gene in the pmirGLO luciferase vector (Promega, USA). HEK293T cells were co-transfected with the pmirGLO reporter plasmids and miR-143-3p mimics or miR-143-3p NC. The relative luciferase activity was measured with the Dual-Luciferase Reporter Assay System (Promega, USA).

### Flow cytometric analysis of apoptosis

The apoptotic assay was conducted using an Annexin V-APC/PI Apoptosis Detection Kit (Multisciences, China) and analyzed with a flow cytometry (BD Biosciences, USA). The ratio of early and late apoptotic cells was detected to calculate the apoptotic rate.

### Isolation of nuclear and cytoplasmic RNA

The isolation of nuclear and cytoplasmic RNA in BGC823 and SGC7901 cells were performed using the PARIS Kit (Invitrogen, USA) according to the manufacturer’s instructions. Then the subcellular fractions were extracted and subjected to qRT-PCR. β-actin (cytoplasm control) and U6 (nucleus control)was used for normalization.

### RNA fluorescent in situ hybridization

A Cy3-labelled CCDC144NL-AS1 complementary DNA probe mix (RiboBio, China) was synthesized in vitro. fluorescent in situ hybridization (FISH) kit (RiboBio, China) was used to detect the subcellular localization of CCDC144NL-AS1 in BGC823 and SGC7901 cells according to the manufacturer’s instructions. Images were observed with a confocal laser-scanning microscope (Olympus FV1000, Japan).

### Immunohistochemistry (IHC)

All specimens were fixed and then embedded in paraffin. The paraffin-embedded sections were de-waxed in xylene and were rehydrated in graded alcohols. Then, specimens were incubated with primary antibodies Ki-67 (Abcam, USA) followed by secondary antibody conjugated with HRP. Subsequently, detection was conducted by 3,3′-diaminobenzidine and haematoxylin. The staining positivity was quantified in three different high-power fields of each section.

### Hematoxylin and eosin staining

Hematoxylin and eosin (HE) was used to stain paraffin-embedded lung sections of mice containing metastases. Sections were cut into 5-μm slices for pathological evaluation and observation under a microscope (Olympus, Japan).

### Statistical analysis

Statistical analyses were performed using SPSS 22.0 (IBM, USA) and GraphPad Prism, version 6.00 (GraphPad Software, USA). Experimental data were shown as mean ± standard deviation (SD),the significance of differences between groups was estimated by the two-tailed student *t*-test, Wilcoxon test, *χ*^2^ test or analysis of variance (ANOVA). Each experiment was repeated independently at least three times, *p* < 0.05 was considered statistically significant.

## Supplementary information

Supplementary Figure S1

Supplementary Figure S2

Supplementary Figure S3

Supplementary Figure S4

Supplementary Figure S5

Supplementary Table 1

Supplementary Table 2

Supplementary Figure and Table Legends
