# Supplementary figures and images for: Long non-coding RNA CCDC144NL-AS1 sponges miR-143-3p and regulates MAP3K7 by acting as a competing endogenous RNA in gastric cancer
Source: Cell Death Dis. 2020 Jul 9;11(7):521. doi: 10.1038/s41419-020-02740-2 (PMC7347562; doi:10.1038/s41419-020-02740-2)

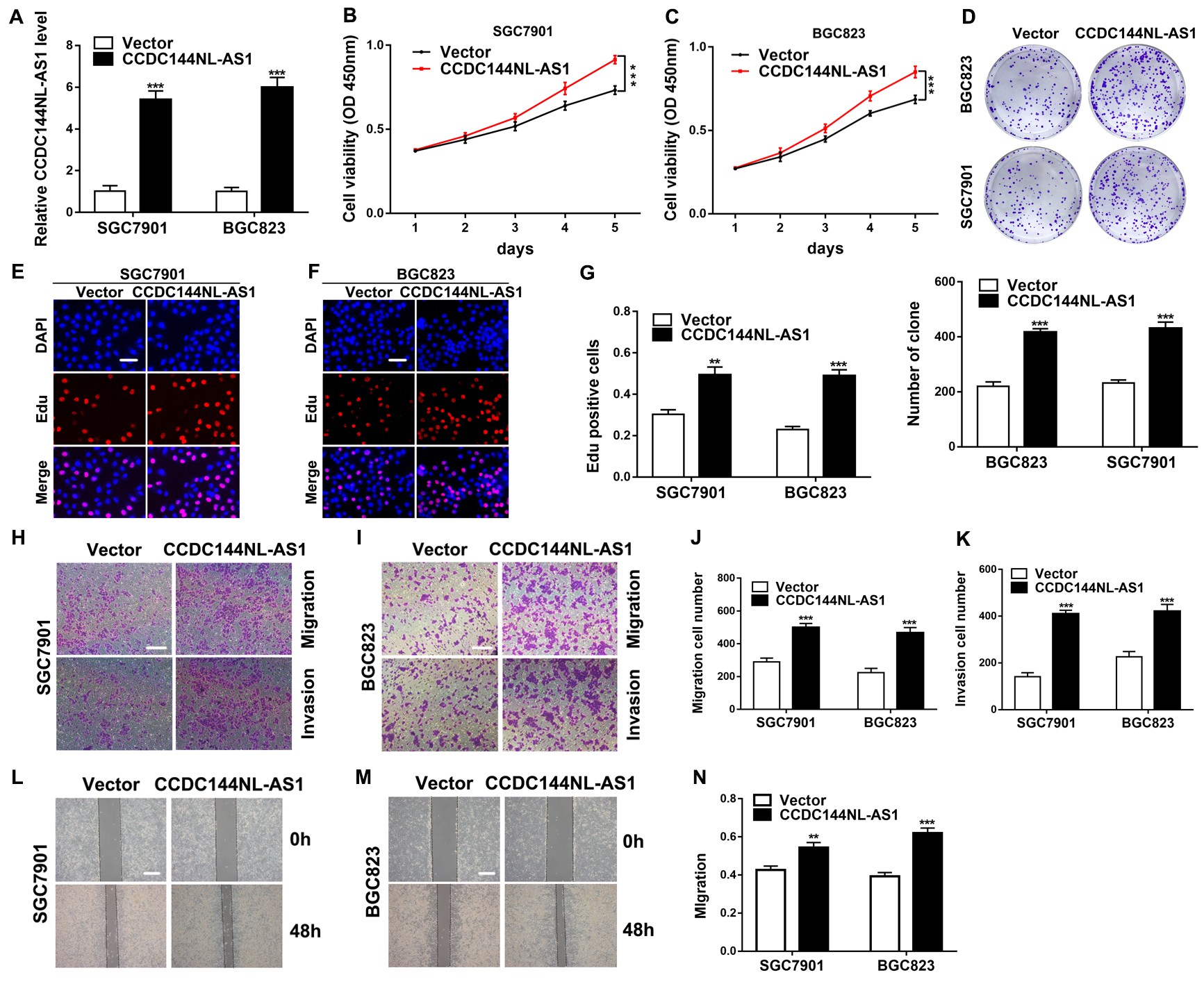

Supplement: Supplementary file 1 — Supplementary Figure S1 [file 41419_2020_2740_MOESM1_ESM.tif]

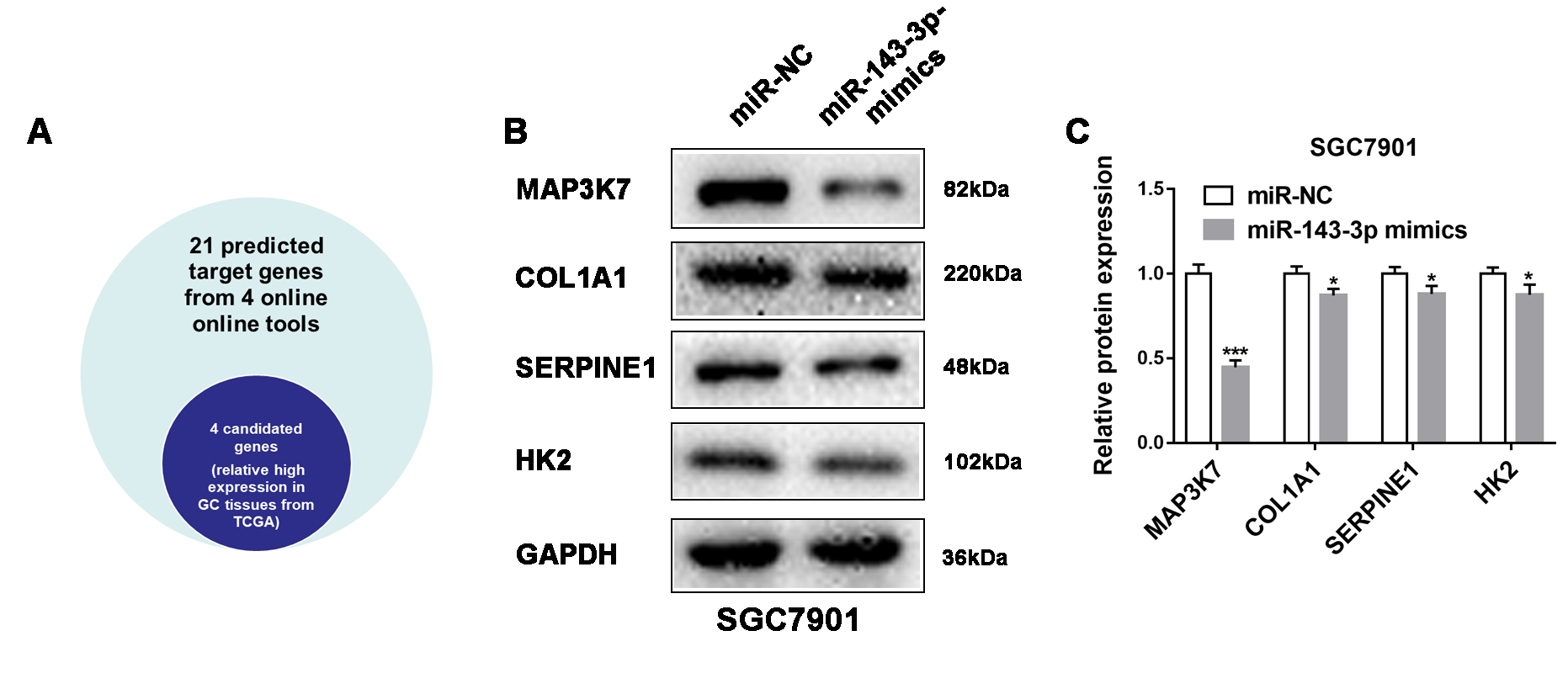

Supplement: Supplementary file 2 — Supplementary Figure S2 [file 41419_2020_2740_MOESM2_ESM.tif]

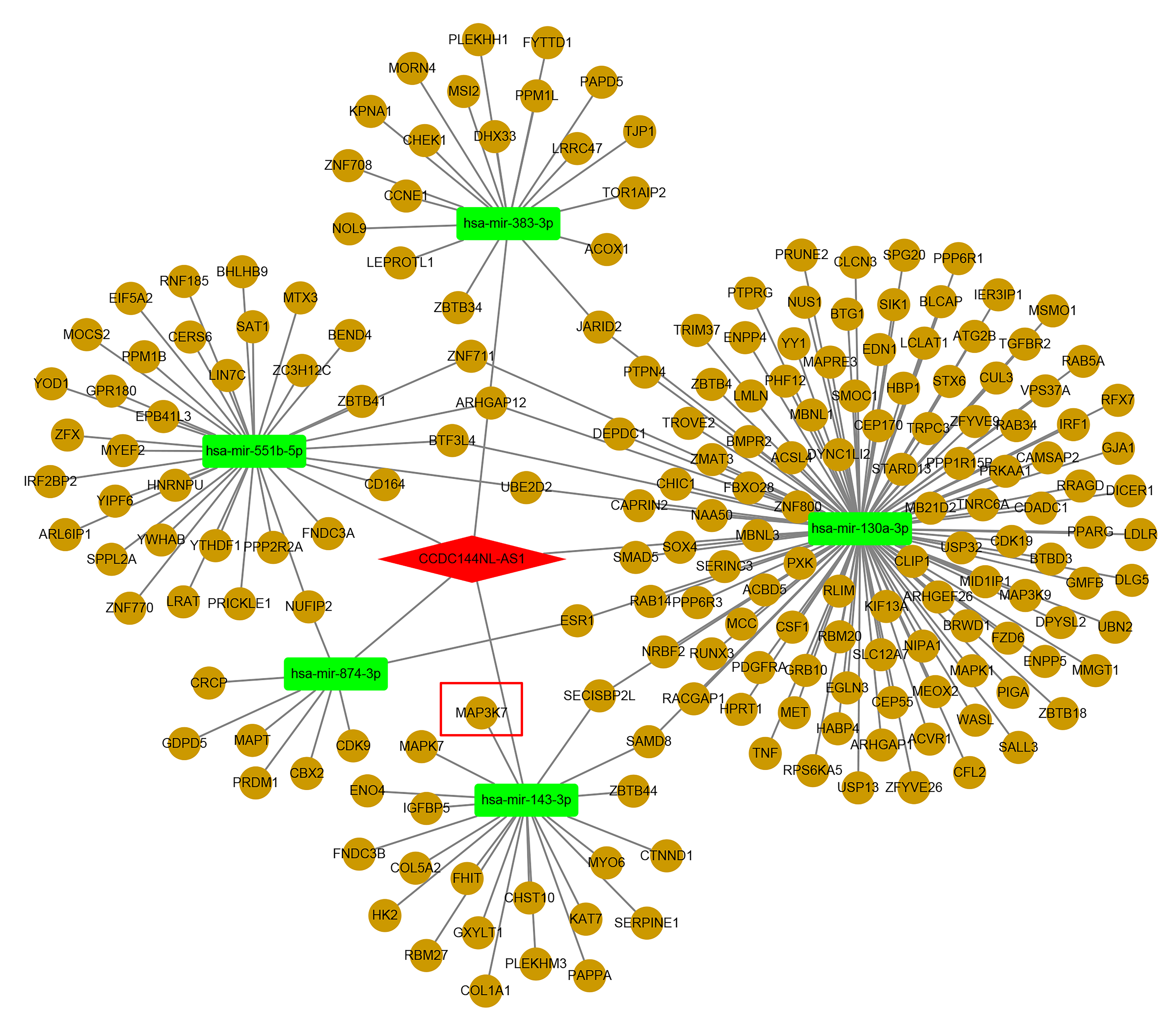

Supplement: Supplementary file 3 — Supplementary Figure S3 [file 41419_2020_2740_MOESM3_ESM.tif]

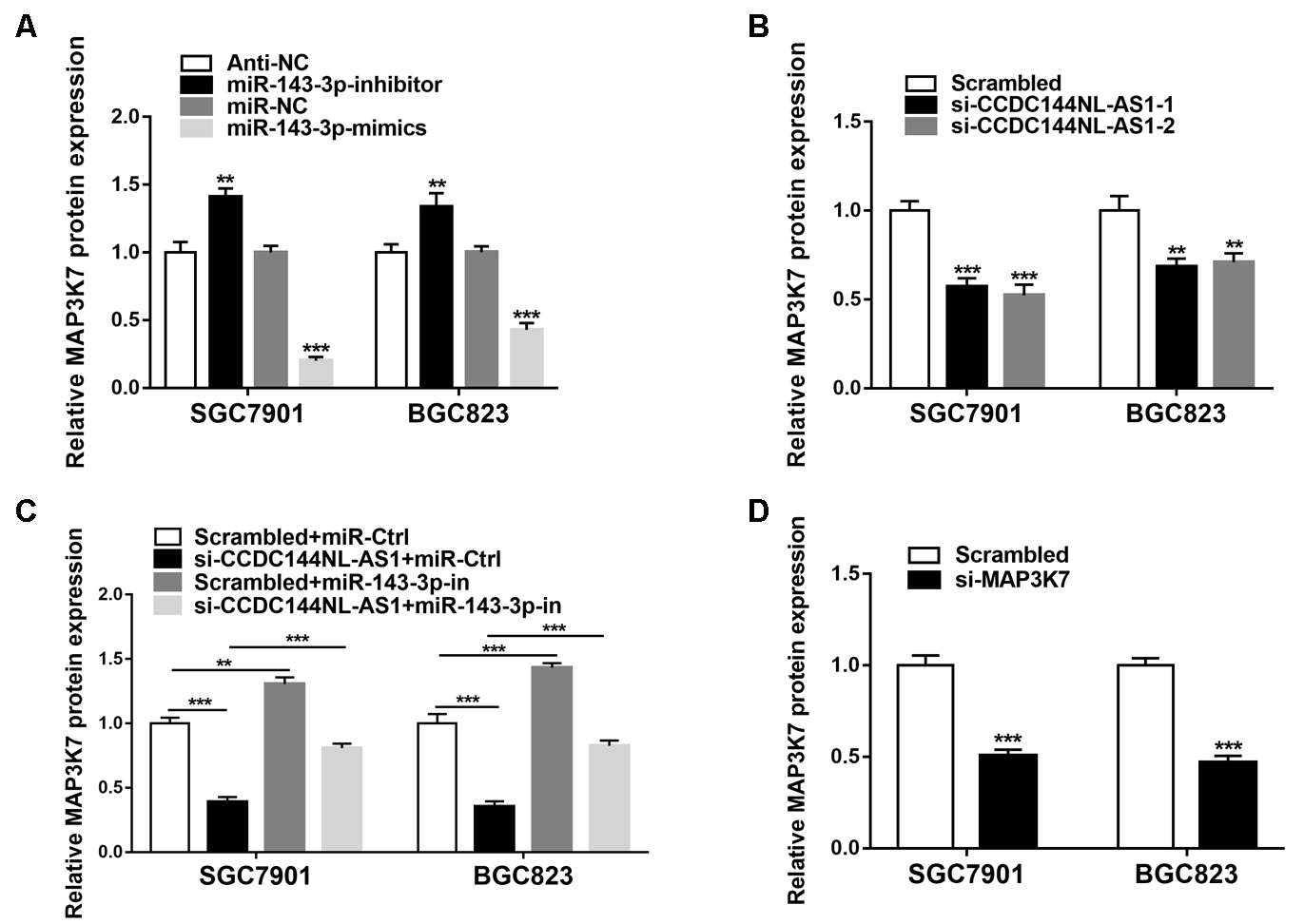

Supplement: Supplementary file 4 — Supplementary Figure S4 [file 41419_2020_2740_MOESM4_ESM.tif]

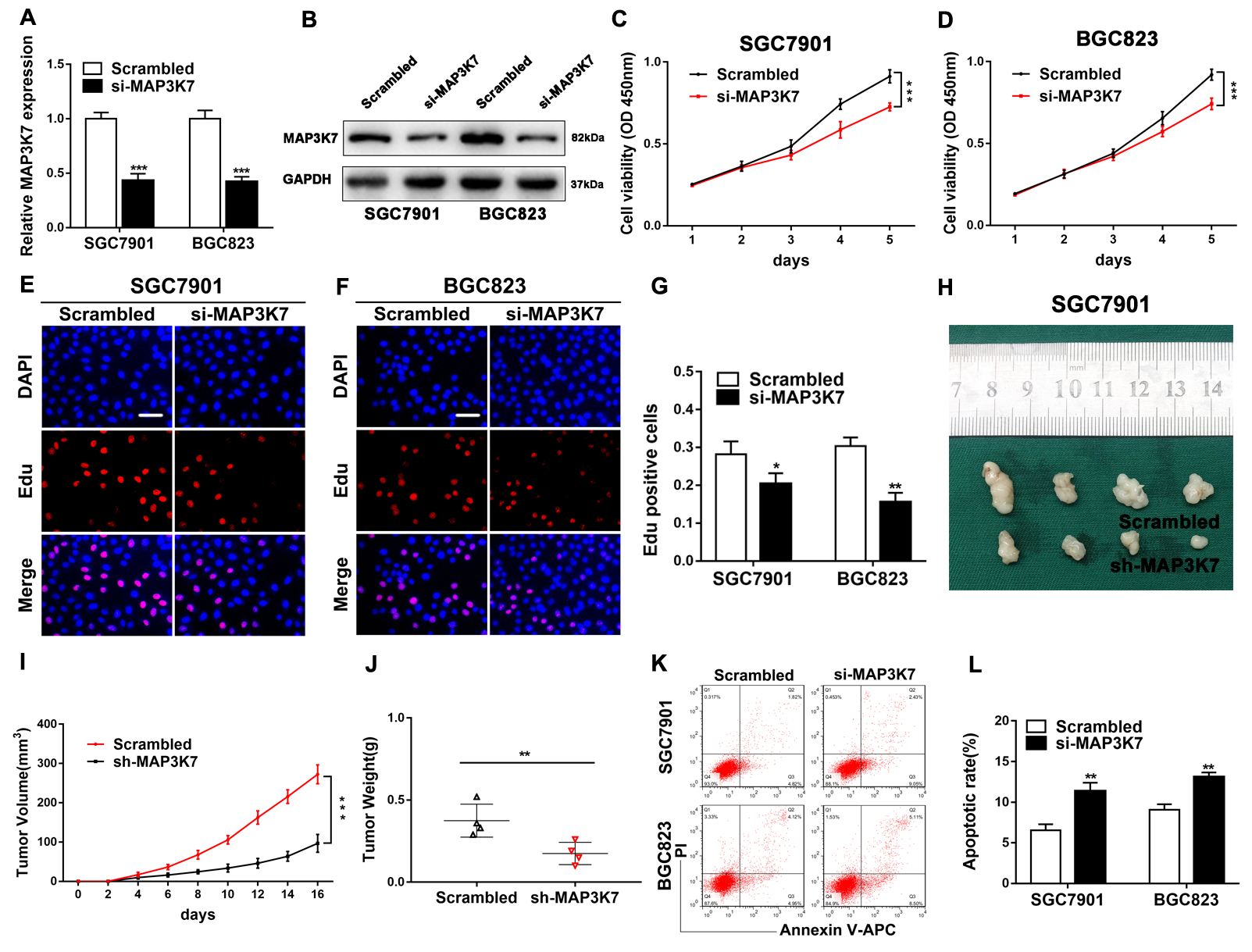

Supplement: Supplementary file 5 — Supplementary Figure S5 [file 41419_2020_2740_MOESM5_ESM.tif]
