# Supplementary material for: Long non-coding RNA CCDC144NL-AS1 sponges miR-143-3p and regulates MAP3K7 by acting as a competing endogenous RNA in gastric cancer
Source: Cell Death Dis. 2020 Jul 9;11(7):521. doi: 10.1038/s41419-020-02740-2 (PMC7347562; doi:10.1038/s41419-020-02740-2)
Supplement: Supplementary file 6 — Supplementary Table 1 [file 41419_2020_2740_MOESM6_ESM.docx]

| Characteristics | Number | CCDC144NL-AS1 expression | | **P-value** |
| --- | --- | --- | --- | --- |
|  |  | **High** | **Low** |  |
| Age(years) |  |  |  |  |
| <60 | 25 | 11 | 14 | 0.458 |
| ≥60 | 47 | 25 | 22 |  |
| **Gender** |  |  |  |  |
| Male | 49 | 24 | 25 | 0.800 |
| Female | 23 | 12 | 11 |  |
| **Size** |  |  |  |  |
| <3 | 29 | 10 | 19 | **0.031*** |
| ≥3 | 43 | 26 | 17 |  |
| **Differentiation** |  |  |  |  |
| Well+Moderate | 35 | 14 | 21 | 0.099 |
| Poor+Undifferentiated | 37 | 22 | 15 |  |
| **Lymphatic metastasis** |  |  |  |  |
| Yes | 38 | 25 | 13 | **0.005*** |
| No | 34 | 11 | 23 |  |
| **Invasion depth** |  |  |  |  |
| T1+T2 | 34 | 15 | 19 | 0.345 |
| T3+T4 | 38 | 21 | 17 |  |
| **TNM stage** |  |  |  |  |
| I+II | 41 | 18 | 23 | 0.234 |
| III+IV | 31 | 18 | 13 |  |

Supplementary table 1. Correlation between CCDC144NL-AS1 expression and clinicopathological characteristics of gastric cancer patients.

*P<0.05 was considered significant
