# Supplementary material for: Long non-coding RNA CCDC144NL-AS1 sponges miR-143-3p and regulates MAP3K7 by acting as a competing endogenous RNA in gastric cancer
Source: Cell Death Dis. 2020 Jul 9;11(7):521. doi: 10.1038/s41419-020-02740-2 (PMC7347562; doi:10.1038/s41419-020-02740-2)
Supplement: Supplementary file 7 — Supplementary Table 2 [file 41419_2020_2740_MOESM7_ESM.docx]

Supplementary table 2. Sequences and reagents used in this study

| **qRT-PCR (5'-3')** |  |
| --- | --- |
| CCDC144NL-AS1-Forward | AGCTGGAGATCACTTAGTGTAAGG |
| CCDC144NL-AS1-Reverse | AAGCTAGGCTTGTCTTTATTCCT |
| miR-143-3p-Forward | TGAGATGAAGCACTGTAGCTC |
| miR-130a-3p-Forward | CAGTGCAATGTTAAAAGGGCAT |
| miR-874-3p-Forward | CTGCCCTGGCCCGAGGGACCGA |
| miR-551b-5p-Forward | GAAATCAAGCGTGGGTGAGACC |
| miR-383-3p-Forward | ACAGCACTGCCTGGTCAGA |
| Universal | GCGAGCACAGAATTAATACGAC |
| MAP3K7-Forward | CCGGTGAGATGATCGAAGCC |
| MAP3K7-Reverse | GCCGAAGCTCTACAATAAACGC |
| β-actin-Forward | GGGAAATCGTGCGTGACATTAAGG |
| β-actin -Reverse | CAGGAAGGAAGGCTGGAAGAGTG |
| U6-Forward | CTCGCTTCGGCAGCACA |
| U6-Reverse | AACGCTTCACGAATTTGCGT |
| **siRNA/shRNA** |  |
| si-CCDC144NL-AS1#1 | GGAAUUGGUGAUUGGCUUUTT |
| si-CCDC144NL-AS1#2 | CCUGUACAUCCUUACCUAUTT |
| sh-CCDC144NL-AS1 | GTGTGGGAAGCTATAAGCATT |
| si-MAP3K7 | UGGCUUAUCUUACACUGGA |
|  | CACCGGACATTGCTTCTACAAATACTCAAGAGGTAT |
| sh-MAP3K7 | TTGTAGAAGCAATGTCC |
| **miRNA** |  |
| miR-143-3p-mimics | UGAGAUGAAGCACUGUAGCUC |
|  | GCUACAGUGCUUCAUCUCAUU |
| mimics NC | UUCUCCGAACGUGUCACGUTT |
|  | ACGUGACACGUUCGGAGAATT |
| miR-143-3p-inhibitor | GAGCUACAGUGCUUCAUCUCA |
| inhibitor NC | CAGUCAUUUUGUGUAGUACAA |
| **Antibody** |  |
| MAP3K7 | 4505, Cell Signaling Technology |
| COL1A1 | 91144, Cell Signaling Technology |
| SERPINE1 | 11907, Cell Signaling Technology |
| HK2 | ab209847, Abcam |
| GAPDH | [5174](https://www.cst-c.com.cn/products/primary-antibodies/gapdh-d16h11-xp-rabbit-mab/5174?site-search-type=Products), Cell Signaling Technology |
