# Supplementary material for: Long non-coding RNA CCDC144NL-AS1 sponges miR-143-3p and regulates MAP3K7 by acting as a competing endogenous RNA in gastric cancer
Source: Cell Death Dis. 2020 Jul 9;11(7):521. doi: 10.1038/s41419-020-02740-2 (PMC7347562; doi:10.1038/s41419-020-02740-2)
Supplement: Supplementary file 8 — Supplementary Figure and Table Legends [file 41419_2020_2740_MOESM8_ESM.docx]

**Long non-coding RNA CCDC144NL-AS1 sponges miR-143-3p and regulates MAP3K7 by acting as a competing endogenous RNA in gastric cancer**

Hao Fan^1^, Yugang Ge^1^, Xiang Ma^1^, Zengliang Li^1^, Liang Shi^1^, Linling Lin^1^, Jian Xiao^1^, Wangwang Chen^1^, Peidong Ni^1^, Li Yang^1,2^, Zekuan Xu^1^

^1^ Department of General Surgery, the First Affiliated Hospital of Nanjing Medical University, Nanjing, Jiangsu Province, China

^2^ Department of General Surgery, Liyang People’s Hospital, Liyang Branch Hospital of Jiangsu Province Hospital, Liyang, Jiangsu Province, China

**Supplementary Figure and Table Legends**

Supplementary Figure 1.

A. Relative expression of CCDC144NL-AS1 confirmed by qRT-PCR in SGC7901 and BGC823 cells with CCDC144NL-AS1 overexpression. B,C. CCK-8 assay was applied to detect cell viability. D-G. Colony formation assays and the Edu assays were conducted to measure cell proliferation ability (scale bar: 100 μm for Edu assay). H-N, Representative results of transwell assays and wound healing assays of cells after overexpression of CCDC144NL-AS1 (scale bar: 200 μm for transwell assay, 100 μm for wound healing assay). **p < 0.01, ***p < 0.001.

Supplementary Figure 2.

A. 21 target genes that can bind with miR-143-3p were selected out by 4 bioinformatics prediction websites (DIANA, miRDB, miRTarBase and TargetScan) and expression levels from TCGA databases. B,C. The protein levels of 4 candidate target gene was detected after miR-143-3p mimics and NC transfection in SGC7901 cells by western blot assays. *p < 0.05, ***p < 0.001.

Supplementary Figure 3.

The lncRNA-miRNA‑mRNA ceRNA network analysis in gastric cancer. The ceRNA network analysis was constructed by Cytoscape. Diamond, rectangle and circle represented lncRNAs, miRNAs and mRNAs, respectively.

Supplementary Figure 4.

A-D. Quantitative analysis of MAP3K7 protein levels in BGC823 and SGC7901 cells transfected with miR-143-3p inhibitor, miR-143-3p mimics, si-CCDC144NL-AS1, si-CCDC144NL-AS1+miR-143-3p inhibitor, si-MAP3K7 or their control groups. **p < 0.01, ***p < 0.001.

Supplementary Figure 5.

A,B. Relative expression of MAP3K7 confirmed by qRT-PCR and western blot in SGC7901 and BGC823 cells treated with si-MAP3K7. C-G. CCK-8 assays and the Edu assays were conducted to measure cell proliferation ability after knockdown of MAP3K7 (scale bar: 100 μm for Edu assay). H. Xenograft tumors in the nude mouse model under different treatments. I,J. Analysis of tumor size and weight in different groups. K,L. Flow cytometry analysis of GC cells apoptosis rates after transfection. *p < 0.05, **p < 0.01, ***p < 0.001.

Supplementary Table 1.

Correlation between CCDC144NL-AS1 expression and clinicopathological characteristics of gastric cancer patients.

Supplementary Table 2.

Sequences and reagents used in this study.
